# Circulating Spexin Is Associated with Body Mass Index and Fat Mass but Not with Physical Activity and Psychological Parameters in Women across a Broad Body Weight Spectrum

**DOI:** 10.3390/jcm11175107

**Published:** 2022-08-30

**Authors:** Maria Suhs, Andreas Stengel, Amelie Rudolph, Selina Schaper, Ellen Wölk, Peter Kobelt, Matthias Rose, Tobias Hofmann

**Affiliations:** 1 Charité Center for Internal Medicine and Dermatology, Department of Psychosomatic Medicine, Charité–Universitätsmedizin Berlin, Corporate Member of Freie Universität Berlin and Humboldt-Universität zu Berlin, 12203 Berlin, Germany; 2 Department of Psychosomatic Medicine and Psychotherapy, University Hospital Tübingen, 72076 Tübingen, Germany; 3 Quantitative Health Sciences, Outcomes Measurement Science, University of Massachusetts Medical School, Worcester, MA 01655, USA

**Keywords:** gut–brain axis, patient-reported outcome, psychoendocrinology, psychometric, psychosomatic stress

## Abstract

Spexin (SPX) is a novel, widely expressed peptide, with anorexigenic effects demonstrated in animal models and negatively correlated with body mass index (BMI) in humans. It increases locomotor activity in rodents and is elevated in human plasma following exercise. Studies have also shown an effect of stress and anxiety on SPX’s expression in different brain structures in animals. The relationships between plasma SPX and physical activity, body composition, and patient-reported outcomes such as perceived stress, depressiveness, anxiety, and eating behaviors are unknown and were examined in this study over a wide BMI range. A total of 219 female (n = 68 with anorexia nervosa; n = 79 with obesity; n = 72 with normal weight) inpatients were enrolled. Perceived stress (PSQ 20), anxiety (GAD 7), depressiveness (PHQ 9), and eating disorder pathology (EDI 2), as well as BMI, bioimpedance analysis, and accelerometry, were measured cross-sectionally at the beginning of treatment and correlated with plasma SPX levels (measured by ELISA) obtained at the same time. Plasma SPX levels were negatively associated with BMI (r = −0.149, *p* = 0.027) and body fat mass (r = −0.149, *p* = 0.04), but did not correlate with perceived stress, anxiety, depressiveness, eating behavior, energy expenditure, and physical activity (*p* > 0.05). The results replicate the negative correlation of SPX with BMI and fat mass, but do not support the hypothesis that peripheral SPX plays a role in the regulation of stress, depressiveness, anxiety, eating behavior, or physical activity.

## 1. Introduction

Spexin (SPX), also known as Neuropeptide Q, is a novel peptide comprised of 14 amino acids and was described for the first time by Mirabeau et al. in 2007 [1]. SPX mRNA and protein were detected in many different tissues, both in animals and in humans. In rodents and humans, SPX mRNA was found in the central nervous system (e.g., hypothalamus [2], hippocampus) and in peripheral tissues (e.g., stomach, small intestine, liver, pancreas [3], fat and other endocrine tissues [4]). In human subjects, the lowest SPX gene expression was detected in muscle- and connective tissue [4]. The fact that SPX mRNA was identified in several different types of tissues suggests that SPX may be involved in various physiological processes and serve as a pleiotropic peptide.

SPX may be involved in fat tissue metabolism, through increasing lipolysis and inhibiting lipogenesis [5]. Furthermore, a decreased uptake of long-chain fatty acids in adipocytes, in rodents with diet-induced obesity after peripheral SPX administration, suggests that SPX may contribute to weight loss [6]. In line with this observation, several studies involving humans have demonstrated a negative correlation between serum SPX and serum triglyceride levels [4,7], as well as body mass index (BMI) [7]. In addition, higher circulating SPX levels were observed in non-obese compared to obese adults [8,9] and children [10,11], although not all studies seem to support these results (e.g., no correlation between BMI and body fat with serum SPX, as well as no difference in serum SPX level between NW and OB/overweight female adolescents [12]).

Not only body weight, but also feeding behavior, might be influenced by SPX [13]. It was shown that food intake led to an increase in SPX mRNA expression in the hypothalamus of Siberian sturgeons, possibly pointing towards an anorexigenic function of SPX [14]. Moreover, intracerebroventricular injection of SPX in goldfish resulted in downregulation of the expression of the orexigenic peptides neuropeptide Y (NPY), orexin, and Agouti-related protein (AgRP), and in higher expression of anorexigenic peptides such as proopiomelanocortin (POMC), cholecystokinin (CCK), and melanin-concentrating hormone (MCH) [15]. The postulated anorexigenic function of SPX is unlikely to be triggered by taste aversion [6].

Another important function of SPX is its possible role in the response to physical activity (PA). For instance, a study in mice showed increased locomotor activity after intraperitoneal SPX injection [6]. Furthermore, a recent report in humans demonstrated that circulating plasma SPX levels significantly were increased in a group of participants categorized as positive responders to exercise. Following a 3-month exercise program, they showed an increased maximal oxygen consumption (VO_2max_) during exercise and, compared to non-responders who did not show an amelioration of VO_2max_, greater improvement in their metabolic profile (total cholesterol, HbA1c, HOMA-IR) [9]. This may be of relevance, as PA plays an important role in maintaining body weight [16] and, in the form of hyperactivity, it is not only a symptom of anorexia nervosa (AN) [17], but also part of its pathogenesis [18]. In patients with obesity, PA has been shown to be inversely associated with the grade of adiposity [19].

Besides its possible functions in the regulation of metabolism, body weight, and physical activity, SPX may be involved in stress response. For instance, fish exposed to stress showed an increase in SPX mRNA expression in different brain areas (e.g., optic tectum, hypothalamus, and midbrain) [20]. Moreover, it was demonstrated that intrahippocampally injected corticotropin-releasing factor (CRF), which is crucially involved in the stress response, decreases SPX expression in different brain tissues (such as hippocampus, hypothalamus, or pituitary gland) in mice [21]. Additionally, another study in fish found that SPX may influence the serotoninergic system, through the upregulation of serotonin-related genes in the raphe nuclei [22]. Moreover, intraperitoneal administration of escitalopram, a serotonin reuptake inhibitor mostly used for the treatment of major depression and general anxiety disorder, led to the downregulation of SPX mRNA in the hypothalamus and upregulated expression of SPX mRNA in the hippocampus and striatum in rats [23]. Therefore, SPX may also be involved in the regulation of stress, anxiety, and depressiveness.

As some studies on SPX indicated its anorexigenic effects and its role in lipogenesis and PA, we hypothesized that SPX may be a factor involved in energy expenditure, and thus it may be associated with different patterns of PA. Furthermore, we hypothesized that SPX levels might be associated with eating disorder pathology, as well as perceived stress, anxiety, and depressiveness. Therefore, we aimed to further examine the link between plasma SPX levels and body composition and PA along with patient-reported outcomes under naturalistic conditions in an inpatient setting. We studied women across a wide BMI range, to examine the impact of body weight and to control for possible gender differences.

## 2. Materials and Methods

### 2.1. Ethics Statement

All investigations were conducted according to the Declaration of Helsinki and all patients gave written informed consent. The study was approved by the institutional ethics committee of the Charité–Universitätsmedizin Berlin (protocol numbers: EA1/130/16)

### 2.2. Subjects

In the present study, 219 female inpatients (68 with a diagnosis of anorexia nervosa (AN), 79 with obesity (OB), and 72 normal-weight (NW) patients treated for conditions other than eating disorders or obesity, such as adjustment disorders, somatoform disorders, or mild depressive episode) were recruited upon admission to the Department of Psychosomatic Medicine at Charité–Universitätsmedizin Berlin (between February 2012 and July 2018). All patients were at an age of ≥18 years. Current pregnancy or lactation period, malignant disease, treatment with immunomodulatory drugs (e.g., methotrexate, azathioprine, and oral corticosteroids), hypercortisolism, and untreated thyroid dysfunction were exclusion criteria. Moreover, women with psychotic disorders, somatoform or somatic disorders of the gastrointestinal system, and those preceding (e.g., bariatric) surgery of the gastrointestinal system, except for appendectomy and uncomplicated cholecystectomy, were excluded.

### 2.3. Anthropometric Measurements

Study enrolment, including clarification of potential exclusion criteria and blood withdrawal, was conducted within four days of admission. Venous blood samples were taken after an overnight fasting period between 7.00 and 8.00 a.m. Patients were permitted to drink a small amount of water, but were advised not to drink coffee, smoke, or exercise before blood withdrawal. On the same morning each patient’s actual medication, body height, and weight in light underwear were assessed and BMI (kg/m^2^) was calculated. Medications and the presence of comorbidities were recorded at admission and discharge. Participants diagnosed with any of the exclusion criteria during their inpatient treatment were excluded.

### 2.4. Physical Activity and Energy Expenditure Assessment

To assess PA, we used a SenseWear^®^ Pro3 armband (BodyMedia, Inc., Pittsburgh, PA, USA), which is a two-axis accelerometer that calculates PA by measuring skin temperature, near-body ambient temperature, galvanic skin response, and heat flux [24]. PA was analyzed for three consecutive days starting from Friday, which was the day of the blood withdrawal. Data were accepted if inpatients wore the armband for more than 20.5 h for at least two out of the three days, as described previously [25]. The PA of the patients was not restricted by the medical staff while wearing the accelerometer.

Using a generalized proprietary algorithm developed by the producer, the total amount of steps, metabolic equivalents of tasks per day (MET), level of energy expenditure, and exercise activity thermogenesis (EAT) were directly calculated after reading out the data. As EAT, we defined an activity of more than three metabolic equivalents of task (METs), which refers to moderate- and vigorous-intensity activities according to the 2011 Compendium of Physical Activities [26].

The thermic effect of food (TEF) was estimated as comprising 10% of total energy expenditure (TEE) and calculated as TEE × 0.1 [27]. Since resting energy expenditure (REE), required for the calculation of NEAT, cannot be directly determined by the SenseWear^®^ armband, it was estimated using weight-group-specific REE prediction equations provided by Müller et al. [28]. Non-exercise-related activity (NEAT) was calculated using the formula NEAT = TEE −TEF − REE − EAT.

### 2.5. Body Composition Measurements

Bioelectric impedance analysis (BIA) was performed between 10:30 a.m. and 1:00 p.m. on the day of blood withdrawal under standardized conditions in the supine position, after subjects had fasted for at least two hours and had lain for half an hour. Phase angle, fat mass, fat free mass, extracellular mass, and body cell mass were assessed using the equations provided by the manufacturer of the bioelectrical impedance analyzer (Nutrigard-M^®^, Data Input^®^, Darmstadt, Germany).

### 2.6. Laboratory Analyses

Blood was collected in pre-cooled standard EDTA tubes prepared with aprotinin for peptidase inhibition (1.2 Trypsin Inhibitory Unit per 1 mL blood; ICN Pharmaceuticals, Costa Mesa, CA, USA) and immediately submerged in ice. After that, tubes were centrifuged at 4 °C for 10 min at 3000× *g* for plasma separation, which was stored at −80 °C, until further processing. After enough samples were collected, SPX plasma levels were measured using a commercial enzyme-linked immunosorbent assay (ELISA, catalog # EK-023-81, Phoenix Pharmaceuticals^®^, Inc., Burlingame, CA, USA). All samples were processed at once. Intra-assay variability was 7.5% and inter-assay variability was <15%. Measurement was performed in January 2019. Every measurement was performed twice, and a mean value was calculated.

### 2.7. Patient-Reported Outcomes

All study participants were asked to fill in the following self-reported questionnaires: Perceived Stress Questionnaire (PSQ), Generalized Anxiety Disorder-7 (GAD-7), Patient Health Questionnaire depression scale (PHQ-9), and Eating Disorder Inventory-2 (EDI-2). Results obtained between two days before and five days after the respective blood withdrawals were accepted.

PSQ-20 is a revised 20-item German version [29] of the Perceived Stress Questionnaire (PSQ; 30 items) [30] and is applied to evaluate subjectively perceived stress. It provides four subscales: “worries”, “tension”, and “joy” as stress responses, and “demands” as the perception of external stressors. It assesses the subjective experience of stress. Cronbach’s alpha for the total scale was 0.73 and for the subscales 0.86 (“worries”), 0.86 (“tension”), 0.81 (“joy”), and 0.84 (“demands”).

The Generalized Anxiety Disorder Questionnaire (GAD-7) [31] is a part of the Patient Health Questionnaire (PHQ) and an established and widely used 7-item screening instrument for diagnosing general anxiety disorder. It also captures symptoms of social anxiety, posttraumatic stress, and panic disorder. In this study, the German version was used [32]. The Cronbach’s alpha for the current sample was 0.87.

The severity of eating disorder symptoms was evaluated using the Eating Disorder Inventory-2 (EDI-2) [33], which is a widely established tool to assess eating disorder pathology in patients suffering from anorexia and bulimia nervosa. It consists of 64 items and encompasses eight subscales, measuring “drive for thinness”, “bulimia”, “body dissatisfaction”, “ineffectiveness”, “perfectionism”, “interpersonal distrust”, “interoceptive awareness”, and “maturity fears”. In our study, sum scores ranging from zero to 100 were created. Moreover, we employed the German translation of the second version [34] and interpreted the first eight, above-mentioned subscales of the EDI-2. The Cronbach’s alpha for the total scale was 0.96, and for the subscales: 0.91 (“drive for thinness”, “bulimia”, and “body dissatisfaction”), 0.90 (“ineffectiveness”), 0.80 (“perfectionism”), 0.82 (“interpersonal distrust”), 0.83 (“interoceptive awareness”), and 0.73 (“maturity fears”).

To assess the severity of depressive symptoms, we used the German version [35] of the PHQ depression scale (PHQ-9) [36]. It consists of nine items that represent the DSM-IV diagnostic criteria for depressive disorders, and its scores range from zero to 27, with scores of ≥10 indicating major depression with a specificity of 0.92 and sensitivity of 0.80 regarding a meta-analysis [37] of 17 validation studies in different languages. The Cronbach’s alpha for the current sample was 0.86.

### 2.8. Statistical Analyses

All statistical analyses were conducted using IBM SPSS Statistics^®^ Version 27.0.0.0 (IBM^®^ Corp, Armonk, NY, USA).

Three groups were created, according to the medical diagnosis and BMI: an anorexia nervosa group (AN) with women diagnosed with anorexia nervosa (n = 68), an obesity group (OB) consisting of patients with a BMI of ≥30.0 kg/m^2^, and a normal weight group (NW) with a BMI between 18.5 kg/m^2^ and 25.0 kg/m^2^ and without a diagnosed eating disorder.

Regarding the explorative design of this study, we established a cut-off of three standard deviations from the mean SPX level to identify outliers. During data analysis, three outliers (two in the anorexia nervosa group and one in the obesity group) were detected and excluded from further statistical analyses, which resulted in a study population of 219 women.

To investigate differences between the three groups, between-group comparisons were made using the Kruskal–Wallis test for non-parametric and one-way ANOVA for parametric data. To assess the frequency distributions between the groups, an overall chi-squared test was performed. In case of significant differences, pairwise comparisons using a chi-squared test were added. Correlations were assessed using Pearson’s for normally, and Spearman’s analysis for non-normally, distributed data. Due to the exploratory approach, we decided not to perform multiple linear regressions. The correlations and differences between groups were considered significant when *p* < 0.05. Due to the explorative design of the study, no corrections for multiple testing were applied.

## 3. Results

### 3.1. Demographic, Socioeconomic, and Medical Characteristics of the Study Population

Demographic and socioeconomic characteristics, comorbidities, and medication of study participants are outlined in Table 1. The AN group was significantly younger than both the OB (*p* < 0.001) and NW groups (*p* < 0.001; Table 1). By definition, patients with AN displayed a lower BMI than patients with OB (*p* < 0.001) and NW subjects (*p* < 0.001), and the NW group had a lower BMI than the OB group (*p* < 0.001; Table 1). Regarding socioeconomic status, the highest proportion of subjects living in a partnership was observed in the NW group. Furthermore, the OB group showed the lowest level of education, as indicated by a lower rate of university entrance diplomas than AN (*p* < 0.01) and NW (*p* < 0.05) and of any other school-leaving qualification than NW (*p* < 0.01; Table 1). NW women were also less often currently unemployed than OB and AN (*p* < 0.05; Table 1).

As expected, type 2 diabetes mellitus, impaired glucose tolerance, insulin resistance, arterial hypertension, hyperuricemia, and fatty liver disease (*p* < 0.001), as well as hypertriglyceridemia *(p* < 0.01), were more common in patients with OB than in AN and NW (Table 1). No significant differences were found between groups in terms of medication taken, except for antidiabetics other than insulin and DPP-4-antagonists/GLP-1 analogs (mostly metformin), which were more common in OB than NW and AN (*p* < 0.01) and for opioids (*p* < 0.01) and other psychopharmacological medication (*p* < 0.05), which were more common in NW and OB than AN. In the NW group, tricyclic antidepressants were more often prescribed than in the AN group (*p* < 0.05, Table 1).

### 3.2. Body Composition, Physical Activity, Energy Expenditure, and Psychometric Characteristics of the Study Population

Data on body composition were available for 191 (57 AN, 71 OB, 63 NW), accelerometric data for 121 (27 AN, 47 OB, 47 NW), and psychometric questionnaires for 218 (GAD-7), 209 (PHQ-9), 214 (PSQ-20), and 195 (EDI-2) of the 219 women (Table 2).

Patients of all three groups showed significant differences from each other in terms of fat mass, fat free mass, body cell mass, and total body water, with the highest values in OB and the lowest in AN (*p* < 0.001; Table 2). Extracellular mass differed between AN and OB (*p* < 0.001), as well as NW and OB (*p* < 0.001), with higher values in OB (Table 2). Lower values were observed for phase angles in AN compared to OB (*p* < 0.001) and NW (*p* < 0.001; Table 2).

With regard to physical activity, patients with OB performed less steps per day than NW *(p* < 0.05) and AN (*p* < 0.001; Table 2). All three groups differed from each other concerning MET per day and TEE, with the highest MET levels in AN followed by NW and OB (*p* < 0.001); and the highest TEE in OB, followed by NW and AN (*p* < 0.01; Table 2). In NW subjects, NEAT was lower (*p* < 0.001) and EAT higher (*p* < 0.001) than in OB. NEAT was also lower in NW than in AN (*p* < 0.001; Table 2). EAT levels were lower in OB in comparison to AN and NW (*p* < 0.001; Table 2).

As shown in Table 2, there were no differences between groups regarding anxiety (GAD-7; *p* > 0.05) and perceived stress (PSQ-20 including all subscales; *p* > 0.05), while patients with AN exhibited higher depression scores than NW (PHQ-9; *p* < 0.01).

Regarding EDI-2 total scores, patients with AN and OB did not differ from each other (*p* > 0.05) but displayed higher scores than NW subjects (*p* < 0.001; Table 2). All three groups differed from each other in the EDI-2 subscale “body dissatisfaction”, with the highest scores in OB followed by AN and NW (*p* < 0.001; Table 2) and in “interoceptive awareness”, with the lowest scores in NW (*p* < 0.001 vs. AN and OB) and the highest in AN (*p* < 0.05 vs. OB; Table 2). Moreover, the NW group showed the lowest, and AN and OB groups similar scores for the subscales “drive for thinness”, “bulimia” (*p* < 0.001), “ineffectiveness” (*p* < 0.01), “maturity fears”, and “interpersonal distrust” (*p* < 0.05). Furthermore, patients with AN displayed a higher “perfectionism” level than patients with OB (*p* < 0.05) and NW (*p* < 0.001; Table 2).

### 3.3. Spexin Is Negatively Associated with Body Mass Index and Fat Mass but Not with Physical Activity or Energy Expenditure

The results of correlation analyses of body composition, physical activity, and energy expenditure, as well as psychometric parameters, with SPX are presented in Table 3.

**Figure 1:**
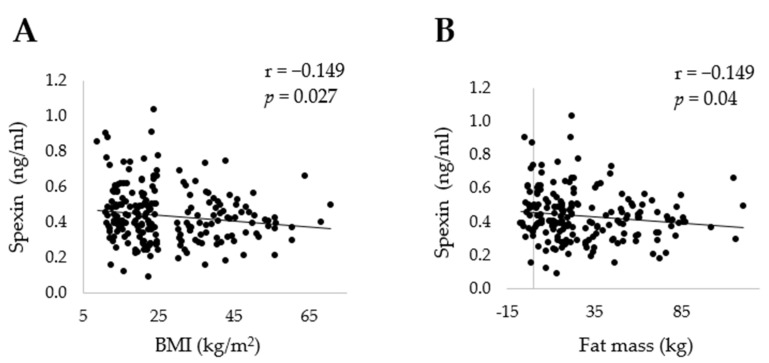
Correlations of spexin with (**A**) BMI and (**B**) fat mass (kg) in the whole study population. Negative fat mass values in bioelectrical impedance analysis are possible in severely underweight patients, due to the manufacturer’s algorithms being calculated primarily for normal weight subjects. Abbreviation: BMI, body mass index.

In the whole study population, the mean plasma SPX concentration was 0.436 ± 0.153 ng/mL (range: 0.092–1.035 ng/mL). SPX levels were found to be significantly higher in AN than OB (*p* < 0.05) and did not differ between the other groups (*p* > 0.05; Table 2). This was reflected by a negative correlation of peripheral SPX with BMI in the whole study group (*r* = −0.149; *p* = 0.027; Figure 1A). Plasma SPX was also negatively associated with absolute (*r* = −0.149; *p* = 0.04; Figure 1B) and relative (*r* = −0.159; *p* = 0.028; Table 3) fat mass. No relationships were observed between circulating SPX and other parameters of body composition, as measured by bioelectrical impedance analysis (*p* > 0.05; Table 3).

We observed no associations between SPX and all measured parameters of physical activity (steps/day, MET/day; *p* > 0.05) and energy expenditure (TEE, EAT, NEAT; *p* > 0.05; Table 3).

### 3.4. SPX Is Not Correlated with Depressiveness, Anxiety, Perceived Stress, and Eating Disorder Pathology in the Whole Study Group

**Figure 2:**
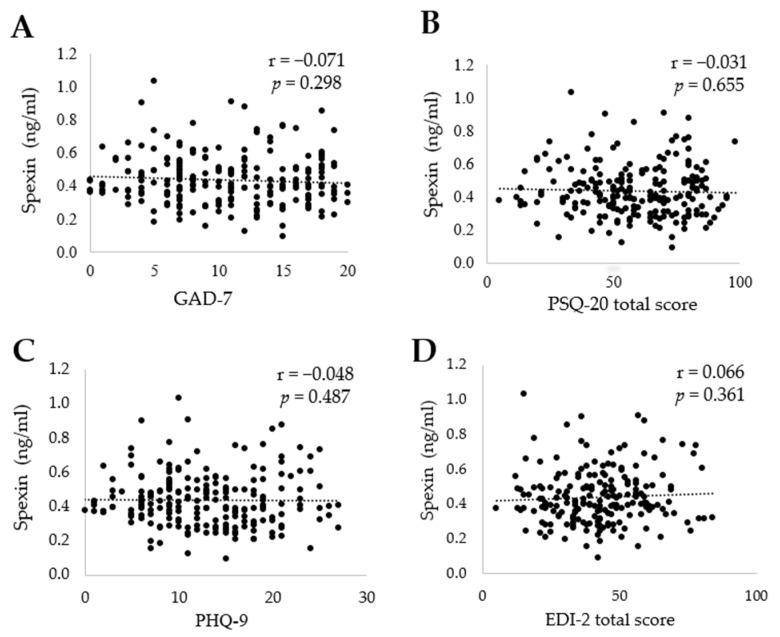
Correlations between (**A**) spexin and anxiety (GAD-7), (**B**) perceived stress (PSQ-20 total score), (**C**) depressiveness (PHQ-9), (**D**) and eating disorder pathology(EDI-2 total score). Abbreviations: EDI-2, Eating Disorder Inventory-2; GAD-7, Generalized Anxiety Disorder-7; PHQ-9, Patient Health Questionnaire-9; PSQ-20, Perceived Stress Questionnaire-20.

No significant associations between SPX and anxiety (GAD-7), depressiveness (PHQ-9), eating disorder pathology (EDI-2), and perceived stress (PSQ-20) total score were observed in the whole study group (Figure 2; Table 3). The PSQ-20 subscale “worries” showed a negative correlation with SPX in the NW group (r = −0.334, *p* = 0.004; Table 3).

## 4. Discussion

The current study investigated the relationship between plasma SPX levels and objectively assessed PA, body composition, and patient-reported outcomes in a group of hospitalized adult women over a wide BMI range. In the whole study population, we showed a weak but statistically significant negative correlation between SPX and BMI, as well as SPX and body fat mass. However, we could not observe any relationships between SPX and parameters of physical activity and SPX and depressiveness, anxiety, stress, and eating disorder psychopathology.

The negative correlation between SPX and body fat mass is in line with earlier observations in adult individuals with obesity, in which a negative association between SPX and body fat percentage was reported [38]. Consistent with this, significantly lower SPX levels were reported in children with high compared to normal fat mass [39]. In one study, no correlation was found between SPX and body fat percentage [12], although this could be explained by the fact that the participants did not differ as much in BMI and body fat percentage as in our study. SPX is reduced after glucose load [4] and leads to lipolysis [5], which could explain the observed negative correlation of SPX with BMI and fat mass, and this would point toward SPX being responsible for the reduction in fat mass and not vice versa and could help to understand SPX’s decrease in the peripheral circulation.

We did not observe any correlations between SPX and eating disorder symptoms as measured by EDI-2. As already mentioned in the introduction, several studies in animal models showed that SPX affects the levels of anorexigenic and orexigenic hormones and its effects result in suppression of food intake through a decrease of orexigenic peptides (AgRP, NPY, orexin) [13,15,40] or upregulation in mRNA expression of anorexigenic peptides (CCK, POMC, MCH) [15], predominantly in the hypothalamus. In addition, a fasting period led to a decline in SPX levels in the forebrain [41], and repeated daily intraperitoneal administration of SPX reduced both the meal size and meal duration, leading to weight loss in animals [6]. However, to date, no study has shown a direct effect of SPX on eating behavior in humans. A study that analyzed the effects of weight gain during inpatient treatment on SPX levels in AN indicated no significant results [42]. Nevertheless, some studies showed a negative association between SPX and leptin [43,44]. Leptin suppresses food intake and leads to weight loss [45]. In blood serum in patients with AN, it is downregulated, which is primarily attributed to the reduced mass of adipose tissue, where peripheral leptin is primarily expressed [46]. Perhaps low leptin also reduces its anorexigenic effects and represents a compensatory mechanism that protects, although insufficiently, against further weight loss [47]. Since both SPX and leptin cause loss of appetite and are simultaneously negatively correlated, it could be claimed that their anorexigenic effects are based on different mechanisms. Given its negative correlation with BMI, SPX, unlike leptin, may act as a driver of weight loss rather than a mere satiety signal and may be one answer to why AN persists or becomes a chronic condition. The fact that SPX was not associated with any scale of the EDI-2 in the present study also suggests that impaired body image or impaired eating habits are not responsible, even partly, for anorexigenic effects of SPX (observed in animals) and the negative association between SPX and leptin (in humans), but that SPX acts predominantly as a brain signal to induce weight loss. Therefore, one might conclude that SPX is not involved in the pathogenesis of AN in terms of impaired eating habits as an expression of eating disorder psychopathology. Longitudinal studies simultaneously measuring leptin and SPX in eating disorders and adjusting for BMI and body fat mass are needed, to further investigate the relationship between SPX and leptin and the function of SPX in eating disorders.

As reported, we also did not observe an association between plasma SPX levels and physical activity, as accelerometrically measured with a SenseWear^®^ armband. This findingdoes not support the findings of a recent study suggesting that SPX could work as an indicator of response to physical activity [9] or correlate with results from an animal study conducted in mice showing increased SPX mRNA expression in muscle tissue and increased concentration in blood serum after exercise [48]. However, a possible explanation for these inconsistent findings could be that, in the present study, PA was measured only cross-sectionally, whereas associations may be detectable only over time or only in subjects responding to exercise. Additionally, SPX might be associated only with voluntary PA, or only in obesity but not in AN and, therefore, might not be associated with the hyperactivity observed in patients with AN. It could also be speculated that the intensity of daily PA, as captured in our study by measuring steps per day, does not increase circulating SPX levels, as in exercise training as reported in men with type 2 diabetes might [49].

We did not identify any association between SPX levels and anxiety (GAD-7), depressiveness (PHQ-9), or perceived stress (PSQ-20). This supports the results of a study conducted in adolescent inpatients with AN, where no associations with depressiveness (measured by BDI), eating disorder symptoms (EAT-26), or obsessive-compulsive disorder symptoms (Y-BOCS) were observed [42]. However, this study did not include any experimental design to investigate the causal relationship between SPX release and psychometric parameters. To date, a possible relationship between (psychological) stress reaction and peripheral SPX has only been demonstrated in animals. One study indicated that overexpression of SPX1 (one of two SPX orthologs occurring in zebrafish) in the dorsal habenula reduced anxiety-associated behaviors in zebrafish [22]. In addition, in mice in which anxiety was induced, SPX mRNA expression was reduced in the hippocampus, whereas CRF mRNA expression was upregulated [21]. Furthermore, CRF treatment has been shown to decrease SPX expression [21], and fish chronically stressed by social defeat exhibited upregulated cortisol and SPX levels in the brain [20]. Thus, studies in animals indicate a role for SPX in the regulation of stress, emotion, and behavior, which may also apply to humans. Therefore, further investigations of changes in peripheral SPX levels following interventions inducing stress or anxiety, e.g., by using stress paradigms such as the Trier social stress test, are needed. However, the investigation of alterations of cerebral SPX expression in humans would require using molecular imaging for peptide detection (such as the nanoflow liquid chromatography-mass spectrometry in combination with invasive microdialysis or the less invasive, but requiring the use of radioactive substances, positron emission tomography [50]). Nonetheless, at present, the known peptide monitoring methods are very expensive and not widespread. Moreover, the fact that repeated imaging would be needed (at least once before and after the stress test) makes the above-mentioned imaging techniques even more difficult to perform. In our explorative study, we did not find any association between PA and stress, and SPX, nor in the whole study group or in the subgroups (data not shown). Unfortunately, no studies investigated the effects of SPX on stress-mediated PA. Since there is evidence in the literature that stress interferes with PA [51], we suggest conducting an interventional study investigating changes in PA patterns and stress-like-behaviors (e.g., using an elevated-zero-maze test) after peripheral or central SPX injection.

The generalizability of the reported results is subject to certain limitations. First, BIA and the SenseWear^®^ armband device are well-established measures for the determination of body composition and physical activity in clinical practice and research. However, BIA might have limited validity in severely underweight subjects [52], and the SenseWear^®^ armband seems to slightly underestimate step counts, so the results must be interpreted with caution [53]. Second, the present study was conducted under naturalistic conditions; therefore, no healthy control group was employed. Consequently, we were unable to compare study participants with and without mental disorders. However, the included patients showed a wide range of psychological impairment on the different scales, so that circulating SPX levels could be well related to the constructs of depressiveness, anxiety, stress, and eating disorder pathology. In addition, the naturalistic design is also a strength, since it reproduces real-world conditions during inpatient treatment. Third, the naturalistic study design entails heterogeneity with regard to existing comorbidities, which are potential confounders and could therefore have contributed to the weak or absent associations observed. Therefore, future studies with more stratified study populations and an experimental research design should be conducted. Fourth, a cross-sectional study can only show associations and not cause-effect relationships. Therefore, in addition to experimental studies with healthy control groups, longitudinal studies are needed, to further examine changes in peripheral SPX levels over the course of improvements under treatment. Lastly, while studies in animals and humans indicate interrelations between SPX and the reproductive system [54], we did not assess menstrual status, and the intake of estrogen-containing medications was not an exclusion criterion in our female study population.

In this exploratory study, our findings replicate the negative association between SPX and both BMI and body fat. However, using a naturalistic and cross-sectional study design, no associations between circulating SPX and both patient-reported outcomes and PA were observed. Since animal studies indicated a possible effect of SPX on PA, anxiety, depressiveness, stress, and feeding behavior, further research in humans, employing longitudinal and interventional studies in more homogenous and larger study samples, is required.

## Figures and Tables

**Table 1:** Demographic and socioeconomic characteristics, comorbidities, and medication of study patients.

Parameter	All Subjects (n = 219)	AN (n = 68)	OB (n = 79)	NW (n = 72)	Significance
Demographic characteristics					
Age (years)	40.7 ± 16.4 (18–85)	28.4 ± 10.4 (18–59)	46 ± 15.3 (19–85)	46.5 ± 16.1 (21–82)	*** ###
Body mass index (kg/m^2^)	27.1 ± 13.3 (8.7–70.7)	14.3 ± 2 (8.7–18.9)	42.3 ± 9.5 (30.1–70.7)	22.2 ± 1.8 (18.6–24.9)	*** ### +++
Socioeconomic characteristics					
Living in a partnership	109 (50%)	23 (34%)	39 (50%)	47 (65%)	### +
Level of education					
University entrance diploma	67 (31%)	28 (41%)	15 (19%)	24 (33%)	** +
Vocational diploma	20 (9%)	4 (6%)	9 (11%)	7 (10%)	
Secondary education certificate	94 (43%)	27 (40%)	32 (41%)	35 (49%)	
Basic school qualification	24 (11%)	6 (9%)	13 (16%)	5 (7%)	
No school-leaving qualification	14 (6%)	3 (4%)	10 (13%)	1 (1%)	++
Currently unemployed	91 (42%)	34 (50%)	36 (46%)	21 (29%)	# +
Unemployment during past 5 years	56 (26%)	18 (26%)	22 (28%)	16 (22%)	
Comorbidities					
Type 2 diabetes mellitus	18 (8%)	0 (0%)	17 (22%)	1 (1%)	*** +++
Impaired glucose tolerance	26 (12%)	1 (1%)	24 (30%)	1 (1%)	*** +++
Insulin resistance	41 (19%)	0 (0%)	36 (46%)	5 (7%)	*** +++
Arterial hypertension	53 (24%)	0 (0%)	38 (48%)	15 (22%)	*** ### +++
Hypercholesterinemia	71 (32%)	11 (18%)	37 (47%)	23 (33%)	*** #
Hypertriglyceridemia	14 (6%)	1 (3%)	12 (15%)	1 (1%)	** ++
Hyperuricemia	22 (10%)	1 (1%)	19 (24%)	2 (3%)	*** +++
Coronary heart disease	4 (2%)	0 (0%)	4 (5%)	0 (0%)	
Fatty liver disease	24 (11%)	1 (1%)	22 (28%)	1 (1%)	*** +++
Degenerative diseases of the musculoskeletal system	72 (33%)	2 (3%)	42 (53%)	28 (39%)	*** ###
Medication					
Insulin	4 (2%)	0 (0%)	4 (5%)	0 (0%)	
DPP-4 antagonists/GLP-1 analogs	2 (1%)	0 (0%)	2 (3%)	0 (0%)	
Other antidiabetics	9 (4%)	0 (0%)	9 (11%)	0 (0%)	** ++
Antipsychotics	26 (12%)	9 (13%)	11 (14%)	6 (8%)	
SSRI/SNRI	45 (21%)	11 (16%)	20 (25%)	14 (19%)	
Tricyclic antidepressants	25 (11%)	3 (4%)	10 (13%)	12 (17%)	#
Other antidepressants	13 (6%)	3 (4%)	6 (7%)	4 (6%)	
Tranquilizers, sedatives, hypnotics	4 (2%)	1 (1%)	1 (1%)	2 (3%)	
Other psychopharmacological medication	12 (5%)	0 (0%)	7 (9%)	5 (7%)	* #
Opioids	19 (9%)	0 (0%)	10 (13%)	9 (13%)	** ##

Data are expressed as absolute numbers with percentages in parentheses. Differences between groups were assessed using Kruskal–Wallis (age and BMI) and χ^2^ tests. Significant differences (without correction for multiplicity) between the AN and OB groups are displayed as * (*p* < 0.05), ** (*p* < 0.01), or *** (*p* < 0.001); between the AN and NW groups as # (*p* < 0.05), ## (*p* < 0.01), or ### (*p* < 0.001), and between the NW and OB groups as + (*p* < 0.05), ++ (*p* < 0.01), or +++ (*p* < 0.001). Abbreviations: AN, anorexia nervosa; DPP-4, dipeptidyl peptidase-4 inhibitor; GLP-1, glucagon-like peptide-1; NW, normal weight; OB, obesity; SSRI, selective serotonin reuptake inhibitors; SNRI, serotonin-norepinephrine reuptake inhibitors.

**Table 2:** Endocrine parameters, body composition, physical activity, energy expenditure, and patient-reported outcomes of the study populations.

Parameter	n	AN	n	OB	n	NW	Significance
Endocrine parameter							
Plasma spexin (ng/mL)	68	0.47 ± 0.16 (0.12–0.90)	79	0.41 ± 0.13 (0.16–0.74)	72	0.43 ± 0.17 (0.09–1.04)	*
Bioelectrical impedance analysis							
Fat mass (kg)	57	2.0 ± 4.4 (−7.0–11.9)	71	58.7 ± 21.4 (30–120.5)	63	18.2 ± 4.8 (10.9–31.1)	*** ### +++
Fat mass (%)	57	3.4 ± 11.3 (−25.4–27.8)	71	49.9 ± 6.2 (37.8–62)	63	29.0 ± 5.2 (19.7–41.7)	*** ### +++
Total body water (L)	57	28.0 ± 3.9 (19.4–38)	71	41.1 ± 6.2 (30.6–57.2)	63	32.2 ± 3 (26.1–40.5)	*** ### +++
Phase angle	57	4.2 ± 1 (1.4–6.1)	71	5.5 ± 0.7 (2.9–7.0)	63	5.3 ± 0.7 (3.3–6.5)	*** ###
Fat free mass (kg)	57	38.3 ± 5.3 (26.5–51.9)	71	56.1 ± 8.5 (41.8–78.1)	63	44.0 ± 4.1 (35.7–55.3)	*** ### +++
Extracellular mass (kg)	57	22.4 ± 5.1 (5.9–41.9)	71	28.6 ± 4.6 (21.0–40.2)	63	22.9 ± 3.1 (17.3–36.2)	*** +++
Body cell mass (kg)	57	15.7 ± 4 (4.1–22.2)	71	27.6 ± 4.8 (16.7–39.2)	63	21.1 ± 2.4 (15.9–26.5)	*** ### +++
Accelerometric measurement							
Number of steps/day	27	11,820 ± 7090 (1736–37,750)	47	7511.4 ± 3306.3 (1344–17,540)	47	9192 ± 3320 (2797–18,897)	*** +
MET/day	27	1.7 ± 0.2 (1.4–2.4)	47	1.1 ± 0.1 (0.8–1.5)	47	1.4 ± 0.2 (1.1–1.7)	*** ### +++
TEE (kcal/kg/day)	27	1715 ± 258 (1277–2427)	47	2961.2 ± 674.9 (1953.6–4753.8)	47	2055 ± 220 (1527–2661)	*** ## +++
EAT (kcal/kg/day)	27	139.9 ± 87.1 (5.0–400)	47	65.3 ± 44.2 (0–202)	47	100.6 ± 47.1 (22–219)	*** +++
NEAT (kcal/kg/day)	27	655.5 ± 127.9 (463–998)	47	694.9 ± 313.7 (63–1575)	47	411.2 ± 132.7 (119–800)	### +++
Patient-reported outcomes							
GAD-7	67	12.0 ± 4.6 (1–19)	79	10.1 ± 5.8 (0–21)	72	10.4 ± 5.6 (0–21)	
PHQ-9	62	14.6 ± 6.1 (2–27)	75	12.7 ± 6.6 (1–26)	72	11.5 ± 5.8 (0–27)	##
PSQ-20	65	60.6 ± 18.2 (13.3–95)	77	58.4 ± 21 (11.7–98.3)	72	56.2 ± 21.4 (5–90)	
Worries	65	60.2 ± 25.5 (0–100)	77	57.7 ± 26.8 (0–100)	72	51 ± 27.5 (0–100)	
Tension	65	70.9 ± 22.1 (20–100)	77	64.8 ± 26.5 (7–100)	72	63.8 ± 26.5 (0–100)	
Joy	65	33.8 ± 22.4 (0–86.7)	77	38.1 ± 23.2 (0–100)	72	38.7 ± 23.3 (0–100)	
Demands	65	45.1 ± 26.3 (0–93.3)	77	49.1 ± 24.4 (0–100)	72	48.5 ± 28.2 (0–100)	
EDI-2 total	56	48.2 ± 13 (13–80)	69	49.1 ± 13.6 (22–84)	70	31.1 ± 14.2 (5–77)	### +++
Drive for thinness	56	55.1 ± 30.9 (0–97)	69	59 ± 21.3 (14–97)	70	23.2 ± 21.2 (0–86)	### +++
Bulimia	56	22.4 ± 23 (0–91)	69	24.3 ± 21.7 (0–94)	70	6.9 ± 14.2 (0–91)	### +++
Body dissatisfaction	56	63.1 ± 18.7 (4–100)	69	83.6 ± 16.1 (47–100)	70	39.7 ± 23.1 (0–100)	*** ### +++
Ineffectiveness	56	48.2 ± 18.9 (16–86)	69	46.5 ± 20.9 (6–90)	70	34.8 ± 18.9 (4–86)	### ++
Perfectionism	56	51.4 ± 20.7 (10–100)	69	43.2 ± 22.6 (3–97)	70	37 ± 22.8 (0–87)	* ###
Interpersonal distrust	56	47.9 ± 18.8 (6–89)	69	47.6 ± 18.8 (6–89)	70	39.3 ± 17.9 (6–94)	# +
Interoceptive awareness	56	45.6 ± 15.2 (12–86)	69	39.1 ± 17.3 (2–90)	70	26.7 ± 16.1 (4–64)	* ### +++
Maturity fears	56	48.7 ± 19.2 (10–90)	69	45.1 ± 17.6 (10–100)	70	39 ± 17.2 (8–78)	## +

Data are expressed as mean ± standard deviation and range in parentheses. Differences between groups were assessed using a Kruskal–Wallis test for non-parametric data and ANOVA for parametric data. Negative fat mass values in bioelectrical impedance analysis are possible in severely underweight patients, due to the manufacturer’s algorithms being calculated primarily for normal weight subjects. Significant differences (without correction for multiplicity) between AN and OB groups are displayed as * (*p* < 0.05), or *** (*p* < 0.001); between AN and NW groups as # (*p* < 0.05), ## (*p* < 0.01), or ### (*p* < 0.001) and between NW and OB groups as + (*p* < 0.05), ++ (*p* < 0.01), or +++ (*p* < 0.001). Abbreviations: AN, anorexia nervosa; EAT, exercise-related activity thermogenesis (energy expenditure of more than three metabolic equivalents of a task); EDI-2, Eating Disorder Inventory-2; GAD-7, Generalized Anxiety Disorder-7; MET, metabolic equivalents of tasks; NEAT, non-exercise-related activity; NW, normal weight; OB, obesity; PHQ-9, Patient Health Questionnaire-9; PSQ-20, Perceived Stress Questionnaire-20; TEE, total energy expenditure.

**Table 3:** Correlations of body mass index, body composition, physical activity, energy expenditure, and patient-reported outcomes with spexin.

Parameter	All Subjects n = 219		Anorexia Nervosa n = 68		Obesity n = 79		Normal Weight n = 72	
	*r*	*p*	*r*	*p*	*r*	*p*	*r*	*p*
Body mass index (kg/m^2^)	−0.149	0.027	−0.204	0.097	0.095	0.404	0.145	0.226
Bioelectrical impedance analysis								
Fat mass (kg)	−0.149	0.04	−0.149	0.27	0.138	0.252	0.198	0.12
Fat mass (%)	−0.159	0.028	−0.15	0.266	0.086	0.473	0.157	0.218
Total body water (L)	−0.082	0.259	0.102	0.451	0.113	0.349	0.14	0.272
Phase angle	−0.095	0.193	−0.111	0.409	0.061	0.613	0.065	0.613
Fat free mass (kg)	−0.084	0.249	0.103	0.446	0.112	0.353	0.135	0.291
Extracellular mass (kg)	−0.037	0.611	0.097	0.474	0.054	0.654	0.105	0.413
Body cell mass (kg)	−0.089	0.219	−0.042	0.754	0.112	0.354	0.166	0.193
Accelerometric measurement								
Number of steps/day	0.049	0.597	0.258	0.193	−0.067	0.652	−0.044	0.767
MET/day	0.151	0.099	0.252	0.205	−0.034	0.821	−0.178	0.232
TEE (kcal/kg/day)	−0.166	0.069	−0.054	0.788	−0.014	0.925	0.159	0.287
EAT (kcal/kg/day)	0.076	0.41	0.164	0.413	−0.059	0.692	−0.058	0.701
NEAT (kcal/kg/day)	0.021	0.820	−0.083	0.682	−0.103	0.490	0.051	0.733
Patient-reported outcomes								
GAD-7	−0.071	0.298	−0.125	0.312	0.029	0.802	−0.186	0.118
PHQ-9	−0.048	0.487	−0.027	0.836	0.128	0.274	−0.2	0.092
PSQ-20 total	−0.031	0.655	0.008	0.952	0.085	0.464	−0.185	0.12
Worries	−0.12	0.079	−0.078	0.539	−0.004	0.974	−0.334	0.004
Tension	0.018	0.792	−0.02	0.874	0.057	0.621	−0.093	0.436
Joy	−0.023	0.74	−0.046	0.715	−0.197	0.085	0.212	0.073
Demands	−0.018	0.792	0.105	0.404	−0.058	0.617	−0.008	0.946
EDI-2 total	0.066	0.361	−0.024	0.859	0.182	0.135	−0.006	0.961
Drive for thinness	0.022	0.764	−0.156	0.251	0.083	0.499	−0.099	0.417
Bulimia	0.027	0.71	−0.039	0.774	0.084	0.491	−0.004	0.977
Body dissatisfaction	0.005	0.946	−0.045	0.742	0.104	0.397	0.067	0.58
Ineffectiveness	0.029	0.692	−0.069	0.613	0.195	0.109	−0.134	0.268
Perfectionism	0.08	0.264	0.122	0.37	0.044	0.72	0.047	0.696
Interpersonal distrust	0.092	0.202	0.147	0.281	0.196	0.106	0.014	0.909
Interoceptive awareness	0.018	0.802	−0.071	0.604	0.057	0.643	−0.045	0.711
Maturity fears	0.043	0.554	0.173	0.203	0.149	0.22	−0.233	0.053

Correlations were assessed using Pearson’s or Spearman’s analyses. Significant correlations are indicated in bold. Abbreviations: EAT, exercise-related activity thermogenesis (energy expenditure of more than three MET); EDI-2, Eating Disorder Inventory-2; GAD-7, Generalized Anxiety Disorder-7; MET, Metabolic equivalents of tasks; NEAT, non-exercise-related activity; PHQ-9, Patient Health Questionnaire-9; PSQ-20, Perceived Stress Questionnaire-20; TEE, Total energy expenditure.

## Data Availability

The data presented in this study are available on request from the corresponding author. The data are not publicly available due to data privacy.
